# Characterizing the pathotype structure of barley powdery mildew and effectiveness of resistance genes to this pathogen in Kazakhstan

**DOI:** 10.1186/s12870-017-1130-3

**Published:** 2017-11-14

**Authors:** Aralbek Rsaliyev, Zhazira Pahratdinova, Shynbolat Rsaliyev

**Affiliations:** 1 The Research Institute for Biological Safety Problems, Gvardeiskiy, Kordaiskiy Rayon, Zhambylskaya Oblast, Kazakhstan; 2 Kazakh Research Institute of Farming and Crop Science, Almalibak, Karasaisky rayon, Almatinskaya oblast, Kazakhstan

**Keywords:** Barley, Powdery mildew, Resistance genes, Pathotype, Virulence

## Abstract

**Background:**

Powdery mildew of barley is a wind-borne and obligate biotrophic pathogen, which ranks among the most widespread barley pathogens worldwide. However, purposeful research towards studying the structure of the barley powdery mildew populations, of their virulence and of effectiveness of certain resistance genes against the infection was not conducted in Kazakhstan till present time. This paper is the first to describe characteristics of the pathotype structure of *Blumeria graminis* f.sp. *hordei (Bgh)* population and effectiveness of resistance genes in two regions of barley cultivation in the republic.

**Results:**

One hundred and seven isolates of *Bgh* were obtained from seven populations occurring on cultivated barley at two geographically locations in Kazakhstan during 2015 and 2016. Their virulence frequency was determined on 17 differential lines Pallas. All isolates were virulent on the resistance gene *Mla8* and avirulent for the resistance genes *Mla9, Mla1 + MlaAl2, Mla6 + Mla14, Mla13 + MlRu3, Mla7 + MlNo3, Mla10 + MlDu2, Mla13 + MlRu3* and *Mlo-5*. The frequencies of isolates overcoming the genes *Mla3, Mla22, Mlat Mlg + MlCP* and *Mla12 + MlEm2* were 0.0–33.33%, and frequencies of isolates overcoming the genes *Mlra, Mlk, MlLa* and *Mlh* ranged from 10.0 to 78.6%. Based on reactions of differential lines possessing the genes *Mla22, Mlra, Mlk, Mlat, MlLa* and *Mlh*, pathotypes were identified. In total, 23 pathotypes with virulence complexity ranging from 1 to 6 were identified. During both years in all populations of South Kazakhstan and Zhambyl regions pathotypes 24 and 64 mainly prevailed.

**Conclusions:**

Obtained data suggest that low similarity of populations *Bgh* in Kazakhstan to European, African, Australian and South-East Asian populations. The present study provides a foundation for future studies on the pathogenic variability within of *Bgh* populations in Kazakhstan and addresses the knowledge gap on the virulence structure of *Bgh* in Central Asia. Complete effectiveness of the resistance genes, for which no corresponding virulence was found, will allow Kazakhstanean breeders to access many modern barley cultivars that those possessing the resistance effectiveness genes.

## Background

Powdery mildew caused by the biotrophic fungus *Bgh* (syn. *Erysiphe graminis f.sp. hordei*), is one of the most destructive diseases of barley (*Hordeum vulgare* L.) worldwide [1–3]. The disease is especially prevalent in moderate to temperate growing regions where yield losses can reach 40% [4]. Powdery mildew has a number of characteristics that support rapid evolution, such as large numbers of asexual haploid spores, sexual recombination during the growing season, and airborne dispersal over large distances [5].

Barley is the second after wheat most important for Kazakhstan cereal crop with total annual grain yield of more than 2 mln tons per hectare. Spring two-rowed barley represents over 90% of all barleys in the country [6]. Such a low output can be explained by strong pressure from abiotic factors, such as drought, heat, and heavy rains in autumn, and by periodic invasions of devastating barley pathogens [7]. Powdery mildew is one of the most powerful factors affecting barley production in Kazakhstan. Annually the pathogen affects crops of winter and spring barley in Kazakhstan. In recent years, disease epidemics have often been observed in the southern and south-eastern regions [8, 9], indicating an expansive spread in the country. Most of the cultivated commercial varieties of barley lack sufficient resistance to powdery mildew and thus, the disease is of economic importance particularly in locations where conditions are conducive for disease development [9]. Breeding of resistant cultivars of barley is still impeded by high intrapopulation variability of *Bgh* and by the ability of the pathogen to overcome host resistance [1–3]. Consequently finding effective and durable control measures to constrain powdery mildew fungi represents an important challenge in crop protection research.

There are several ways of controlling the disease. The primary one is the use of genetically mildew-resistant cultivars. This is a cheap and environmentally safe method. There is a large number of mapped resistance genes that could provide protection against barley powdery mildew infection. More than 85 race-specific resistance genes for powdery mildew have been identified in barley [1]. Many near-isogenic lines have been developed by backcrossing different resistance gene donors into common genetic background, resulting in, e.g., the Pallas differential set [10]. *Mla*, one of the genetically most thoroughly characterized race-specific loci conferring resistance to powdery mildew, spans 32 known alleles and represents a true allelic series on chromosome 1H [11]. The *Mlo* resistance is a kind of resistance that is unique primarily because it is monogenic [12], although it does not conform to the gene-for-gene system [13], and also because its resistance mechanism differs substantially from that of the other kinds of resistance [1, 12].

Powdery mildew can also be controlled with fungicides but these are ecologically undesirable and their frequent use may speed up the evolution towards resistance to fungicides [14]. The efficacy of using genetically mildew-resistant cultivars depends on pathogen virulence [15]. Additionally, the continuous use of these genes often results in selection in favor of pathotypes with the matching virulence genes in the pathogen population, and therefore in the breakdown of the resistance [16].

Globally, populations of *Bgh* have changed and new and virulent pathotypes have emerged [17–23]. Individual genes of specific resistances differ substantially in their effectiveness against the pathogen population comprising both virulent and avirulent pathotypes [24]. New pathotypes not only cause severe epidemics but substantially limit production of commercial cultivars [17–23]. Despite the increasing importance of powdery mildew in Kazakhstan, the population structure of *Bgh* in this region has not been characterized and so far there were no attempts to determine effectiveness of powdery mildew resistance genes for future application in local breeding programs. This study was therefore undertaken to characterize the virulence structure of populations of *Bgh* in barley-growing regions and identify effective powdery mildew resistance genes in the Republic of Kazakhstan.

## Results

### Virulence frequency

**Fig. 1:**
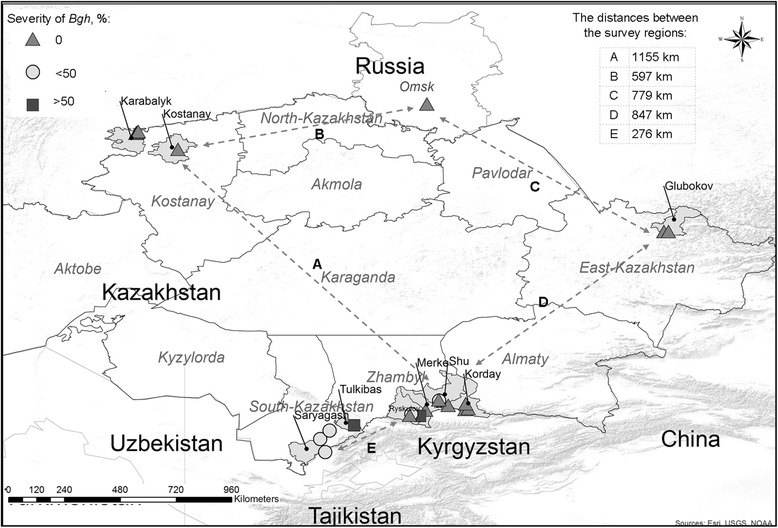
Map showing approximate location of the surveyed regions and distances between them as well as the degree of barley powdery mildew (*Blumeria graminis* f.sp. *hordei*) progress in the regions of Kazakhstan

**Fig. 2:**
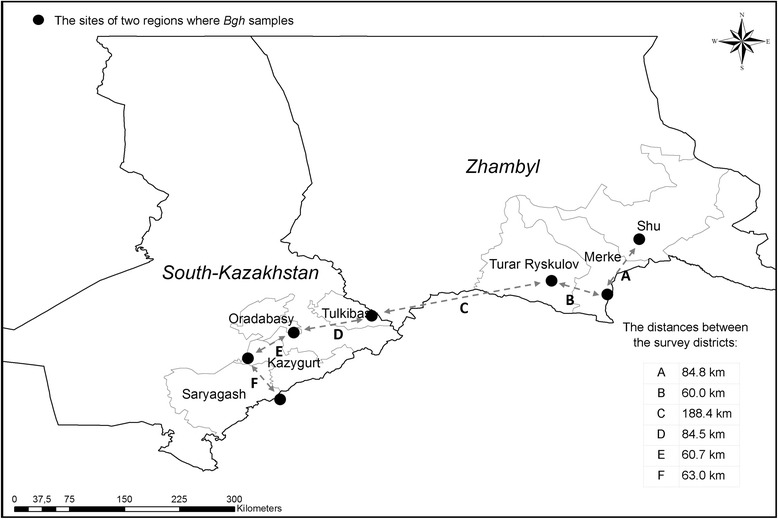
Map showing approximate location of sampling sites in two regions where barley powdery mildew (*Blumeria graminis* f.sp. *hordei*) isolates were collected from 2015 to 2016 to characterize the virulence structure of the pathogen in the Republic of Kazakhstan

In years 2015 and 2016 the isolates of *Bgh* overcoming the resistance genes *Mla9, Mla1 + MlaAl2, Mla6 + Mla14, Mla13 + MlRu3, Mla7 + MlNo3, Mla10 + MlDu2, Mla13 + MlRu3* and recessive gene *Mlo-5* were not found in the surveyed populations (Table 1). Virulent isolates to isogenic lines P02, P21 and P23 with resistance genes *Mla3, Mlg + MlCP* and *Mla12 + MlEm2,* respectively, are also seldom detected: their percentage is 0.9–1.8%. In Kazakhstan virulent isolates to the above mentioned lines were found only in Saryagash population on Yuzhno-Kazakhstanskiy 43 winter barley variety (Table 2, Figs. 1 and 2). In 2 populations (Saryagash, Ordabasy) virulent isolates to gene *Mla22,* and in 3 populations (Saryagash, Tulkubas, Ordabasy) to gene *Mlat* were detected. Occurrence frequency of virulence gene *Vat* differs substantially in the above mentioned populations with the highest percentage in Saryagash population (33.3%). Variation coefficient of occurrence frequency of isolates virulent to resistance genes *Mlra, Mlk* and *MlLa* varied from 10.0 to 78.6%. The isolates virulent to gene *Mlh* were found in all populations with frequency 26.3–71.4% except Ordabasy population. Populations from South Kazakhstan and Zhambyl regions differed in frequency of virulence to lines with genes *Mla22, Mlat, Mla3, Mla12 + MlEm2* and *Mlg + MlCP*. Occurrence frequency of virulence to these genes was 1.5–18.2% in South Kazakhstan populations and 0% in Zhambyl populations. In all populations all tested isolates were virulent to gene *Mla8* (Table 1)*.*

**Table 1:** A set of barley differential cultivars, their genes for resistance to *Blumeria graminis* f.sp. *hordei* and frequency of corresponding virulences (%) found in two regions in Republic of Kazakhstan in 2015 and 2016

No.	Differential cultivar	Resistance gene(s)	ID/Acc. No.^a^	South Kazakhstan region					Zhambyl region				Per year		Total in two regions and years
				Sa^b^	Kg	Tb	Ob	Total	Mk	S	Rk	Total	2015	2016	
1	P12	*Mla22*	NGB4943	33.3	0.0	0.0	33.3	15.2	0.0	0.0	0.0	0.0	21.7	0.0	9.3
2	P14	*Mlra*	NGB4945	38.1	68.8	55.0	66.7	54.5	68.4	78.6	75.0	73.2	54.4	67.2	61.7
3	P16	*Mlk*	NGB4947	28.6	62.5	50.0	22.2	42.4	52.6	42.9	25.0	43.9	39.1	45.9	43.0
4	P20	*Mlat*	NGB4951	33.3	0.0	15.0	11.1	18.2	0.0	0.0	0.0	0.0	19. 6	4.9	11.2
5	P23	*MlLa*	NGB4954	14.3	18.7	10.0	66.7	13.6	52.6	21.4	50.0	41.5	15.2	31.2	14.3
6	P24	*Mlh*	NGB4955	71.4	56.3	65.0	0.0	65.2	26.3	42.6	50.0	36.6	65.2	45.9	54.2
7	P01	*Mla1*	NGB4930	0.0	0.0	0.0	0.0	0.0	0.0	0.0	0.0	0.0	0.0	0.0	0.0
8	P02	*Mla3*	NGB4931	4.8	0.0	0.0	0.0	1.5	0.0	0.0	0.0	0.0	2.2	0.0	0.9
9	P03	*Mla6, Mla14*	NGB4932	0.0	0.0	0.0	0.0	0.0	0.0	0.0	0.0	0.0	0.0	0.0	0.0
10	P04B	*Mla7, Ml(No3)*	NGB4934	0.0	0.0	0.0	0.0	0.0	0.0	0.0	0.0	0.0	0.0	0.0	0.0
11	P08B	*Mla9*	NGB4939	0.0	0.0	0.0	0.0	0.0	0.0	0.0	0.0	0.0	0.0	0.0	0.0
12	P09	*Mla10, MlDu2*	NGB4940	0.0	0.0	0.0	0.0	0.0	0.0	0.0	0.0	0.0	0.0	0.0	0.0
13	P10	*Mla12, MlEm2*	NGB4941	9.5	0.0	0.0	0.0	3.0	0.0	0.0	0.0	0.0	4.3	0.0	1.9
14	P11	*Mla13, MlRu3*	NGB4942	0.0	0.0	0.0	0.0	0.0	0.0	0.0	0.0	0.0	0.0	0.0	0.0
15	P21	*Mlg, MlCP*	NGB4952	4.8	0.0	0.0	0.0	1.5	0.0	0.0	0.0	0.0	2.2	0.0	0.9
16	P22	*Mlo-5*	NGB4953	0.0	0.0	0.0	0.0	0.0	0.0	0.0	0.0	0.0	0.0	0.0	0.0
17	Pallas	*Mla8*	NGB4959	100.0	100.0	100.0	100.0	100.0	100.0	100.0	100.0	100.0	100.0	100.0	100.0

^a^Numbers denote The Nordic Gene Bank (NGB) accession numbers

^b^Districts: Sa - Saryagash; Kg - Kazygurt; Tb - Tulkubas; Ob - Ordabasy; Mk - Merke; S - Shu; Rk - Ryskulov

**Table 2:** Source and number of *Blumeria graminis* f.sp. *hordei* isolates collected from commercial barley cultivares in two geographical regions in Kazakhstan from 2015 to 2016

Region	District	Host cultivar	Growth habit^a^	Year	Number of isolates	Frequency of isolates, %^b^
South Kazakhstan	Saryagash	Yuzhno-Kazakhstanskiy 43	W	2015	21	19.63
	Kazygurt	Saule	S	2015	16	14.95
	Tulkibas	Baisheshek	S	2016	20	18.69
	Ordabasy	Bereke 54	W	2015	9	8.41
Zhambyl	Merke	Baisheshek	S	2016	19	17.76
	Shu	Baisheshek	S	2016	14	13.08
	Ryskulov	Arna	S	2016	8	7.48

^a^W – winter barley, S – spring barley

^b^Calculated as number of isolates in each district as the proportion of the total number of isolates expressed as a percentage

### Pathotypes found

**Fig. 3:**
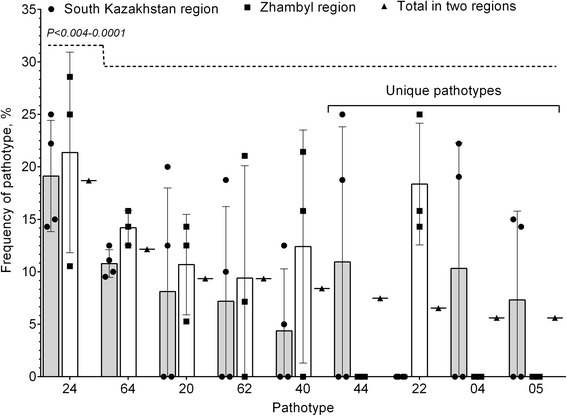
Fluctuation of the most frequent pathotypes in the *Blumeria graminis* f. sp. *hordei* population in two regions of Kazakhstan. Frequency was calculated from the total number of collected and analyzed (for pathotypes) isolates of *B. graminis* f. sp. *hordei* in the populations (Tables 3). Frequency of pathotype 24 occurrence reliably differes from the same of other pathotypes (significant difference from *P < 0.004* to *<0.0001*). Significant difference in occurrence frequency was not observed between other pathotypes (*Р > 0,05*) in the surveyed regions. Statistical analysis was performed using two-way ANOVA followed by Tukey’s multiple comparisons test. *P* values <0.05 were considered significant

In years 2015 and 2016 23 pathotypes of the pathogen were detected among 107 isolates of *Bgh* with the help of the set including six differential cultivars (Table 3): 19 pathotypes in 2015 and 11 in 2016. Seven pathotypes occurred during both years being more frequent in 2016. The majority of the detected pathotypes (14) were original, 4 pathotypes were found in two populations, 1 pathotype in three populations, 2 pathotypes in four populations and 1 pathotype in five populations. Virulence complexity of pathotypes varied from 1 to 6. Wide virulence range (virulence complexity equal to 5–6) was mainly characteristic to unique pathotypes (72, 57, 60) identified only in some populations and represented by single isolates (Tables 3 and 4). In South Kazakhstan and Zhambyl regions 9 pathotypes of the pathogen were prevalent (Fig. 3). Among them 5 pathotypes (24, 64, 20, 62, 40) were detected in two regions, and 4 pathotypes (44, 22, 04, 05) were unique, occurring only in one region. During both years in all populations of South Kazakhstan and Zhambyl regions pathotypes 24 and 64 mainly prevailed at percentage of 19.13–21.37% and 10.78–14.19%, respectively. Some pathotypes also often occurred in different populations, pathotypes 44 and 04 being relatively wide spread in populations of South Kazakhstan region, and pathotypes 40 and 22 – in Zhambyl region (Fig. 3).

**Table 3:** Twenty-three pathotypes of *Blumeria graminis* f.sp. *hordei* found in seven Kazakhstanean populations in 2015 and 2016

Pathotype^a^	Virulence complexity^b^	Number of isolates									
		South Kazakhstan region				Zhambyl region			Per year		Total
		Sa	Kg	Tb	Ob	Mk	S	Rk	2015	2016	
24	3	3	4	3	2	2	4	2	9	11	20
64	4	2	2	2	1	3	2	1	5	8	13
20	2		2	4		1	2	1	2	8	10
62	4		3	2		4	1		3	7	10
40	2		2	1		3	3		2	7	9
44	3		3	5					3	5	8
22	3					3	2	2		7	7
04	2	4			2				6		6
05	3	3		3					3	3	6
02	2					3				3	3
30	3	1			2				3		3
72	6	1							1		1
14	4	1							1		1
54	4	1							1		1
60	5	1							1		1
06	3							1		1	1
57	6				1				1		1
13	4	1							1		1
15	4	1							1		1
43	4	1							1		1
42	3							1		1	1
11	3	1							1		1
21	3				1				1		1

^a^Classification of pathotypes is based on isolate virulence for six differential lines (nos. 1 to 6 in Table 1)

^b^Number of virulences

**Table 4:** Virulence spectra of 23 *Blumeria graminis* f. sp. *hordei* pathotypes in Kazakhstan in years 2015 and 2016

Pathotype	Virulence (+) of the pathotypes to resistance genes:					
	*Mla22*	*Mlra*	*Mlk*	*Mla-t*	*MlLa*	*Mlh*
72	+	+	+		+	
14	+					+
54	+		+			+
60		+	+			
06					+	+
57	+		+	+	+	+
22		+			+	
13	+			+	+	
15	+			+		+
02					+	
30	+	+				
43			+	+	+	
42			+		+	
20		+				
11	+			+		
21		+		+		
04						+
24		+				+
62		+	+		+	
64		+	+			+
40			+			
44			+			+
05				+		+

### Descriptive parameters of populations

Parameters of intrapopulation diversity and statistical indices of difference between assayed populations of *Bgh* are shown in details in Table 5. In total 19 pathotypes were detected in populations of South Kazakhstan region, and nine in populations of Zhambyl region. The greatest number of pathotypes (13) was registered in Saryagash population, and 6–7 pathotypes in each of other populations. Mean of pathotype complexity in populations under study varied from 2.86 (Merke population) to 3.77 (Saryagash), and in two regions this value was equal to 3.48. According to Gleason, Simpson and Shannon indices intrapopulation diversity of phenotypical structure was higher in South Kazakhstan populations (H_G_ – 4.31, D – 0.89, Sh – 2.44) and significantly lower (H_G_ – 2.16, D – 0.78, Sh – 2.03) in Zhambyl populations. Values of these indices in all assayed populations of *Bgh* were as follows: H_G_ – 4.71, D – 0.91 and Sh – 2.76. The greatest richness was observed in Ryskulov population, and the lowest one in Tulkubas population. The total richness of all isolates was just 0.21. However, the highest uniformity of pathotypes allocation was observed in Tulkubas population (1.00) and the lowest one in Shu population (0.95).

**Table 5:** Population parameters of the powdery mildew pathogen in the Republic of Kazakhstan in 2015 and 2016

Population parameter	South Kazakhstan region					Zhambyl region				Total in two regions
	Sa	Kg	Tb	Ob	Total	Mk	S	Rk	Total	
Number of isolates	21	16	20	9	66	19	14	8	41	107
Number of pathotypes	13	6	7	6	19	7	6	6	9	23
Number of pathotypes with at least two isolates	4	6	6	3	10	6	5	2	7	11
Mean of isolate complexity	3.38	3.06	2.95	3.22	3.15	3.00	2.86	3.00	3.95	3.07
Mean of pathotype complexity	3.77	3.00	3.00	3.50	3.63	2.86	3.00	3.00	2.89	3.48
Abundance^a^	19.05	18.75	25.00	22.22	18.18	21.05	28.57	25.00	19.51	18.69
Richness^b^	0.62	0.38	0.35	0.67	0.29	0.37	0.43	0.75	0.22	0.21
*H*_*G*_^*c*^	3.05	1.81	2.01	2.28	4.31	2.04	1.90	2.42	2.16	4.71
*D* ^*c*^	0.85	0.67	0.71	0.67	0.89	0.71	0.67	0.67	0.78	0.91
*H*_*W*_^*c*^	2.46	1.86	1.80	1.71	2.44	1.88	1.72	1.78	2.03	2.76
*E* ^*c*^	0.96	0.96	1.00	0.95	0.86	0.97	0.96	0.99	0.92	0.91

^a^Frequency of the predominant pathotype (%)

^b^Number of pathotypes/number of isolates

^c^ *H*_*G*_ is the Gleason index of diversity, *D* = Simpson index, *H*_*W*_ is the Shannon index of diversity, and *E* is a measure of genetic evenness

## Discussion

Prior to this study, pathogenic variability of *Bgh* populations has been investigated in a number of countries around the globe [17–23]. However, the virulence structure of *Bgh* populations in Kazakhstan and other countries in Central Asia (Uzbekistan, Turkmenistan, Kyrgyzstan, and Tajikistan) was still unknown. This study characterized the virulence structure of *Bgh* populations in barley-growing regions of Kazakhstan and identified pathotypes of *Bgh* and the frequency of their occurrence in two regions of Kazakhstan. To the best of our knowledge, this study represents the first documentation of the virulence structure of *Bgh* in Central Asia.

Based on 107 isolates collected during a 2 year period, 23 pathotypes with virulence complexity ranging from 1 to 6 were identified (Table 3). Two pathotypes, 24 and 64, were identified to be of importance in the barley production in Kazakhstan due to their occurrence across the entire study area. They differ only by one virulence gene, i.e. pathotype 24 is avirulent to gene *Mlk*, and pathotype 64 is virulent. In Kazakhstan all tested isolates were avirulent on lines with genes *Mla9, Mla1 + MlaAl2, Mla6 + Mla14, Mla13 + MlRu3, Mla7 + MlNo3, Mla10 + MlDu2, Mla13 + MlRu3, Mlo-5* and virulent on the line with gene *Mla8* (Table 1). Frequency of isolates virulent to genes*, Mlg + MlCP* and *Mla12 + MlEm2* was very low. In some populations (Saryagash, Tulkubas, Ordabasy) isolates virulent to genes *Mla22* and *Mlat* were detected at frequency of 11.1–33.3%. The isolates virulent to these two genes occurred frequently in European, Australian [19] and South African populations [21]. It has been recently reported that line P12 [10] with gene *Mla22* is the most useful differential cultivar, i.e. possesses the maximal pathogen differentiating ability. However, none of commercial barley varieties includes this gene [20]. In our experiments isolates virulent to genes *Mla3, Mlg + MlCP, Mla12 + MlEm2, Mla22* and *Mlat* were found only in some areas of South Kazakhstan region where winter varieties of barley are cultivated. In total virulence diversity of *Bgh* pathotypes was much higher in populations on winter barley varieties versus spring barley. It has been shown previously that the virulent pathotype (Va22) to gene *Mla22* occurs only on winter barley varieties in France and fails to infect spring barley [25]. In central Europe, winter barley is infected by a broader spectrum of pathogens than spring barley and powdery mildew fungus is dominant among them on susceptible cultivars [26]. Moreover, *Bgh* pathotypes virulent to many resistance genes including genes *Mla3, Mlg + MlCP, Mla12 + MlEm2* и *Mlat* were detected just on winter barley varieties [27]. According to Dreiseitl and Kosman [21] directional selection might be responsible for an increasing virulence frequency to resistance genes *Mla12 + MlaEm2*, *Mlg + MlCP*, and *MlLa*, which may be present in some varieties. Confinement of the most virulent pathotypes (72, 60, 57) to winter varieties of barley (Yuzhno-Kazakhstanskiy 43 and Bereke 54) could sufficiently effect the progress of an epiphytoty, particularly in the areas where these cultivars cover considerable acreages. There is also possibility of their future occurrence and spread on commercial varieties of spring barley. Therefore any strategy based on major resistance genes must incorporate two or more of the following genes *Mla9, Mla1 + MlaAl2, Mla6 + Mla14, Mla13 + MlRu3, Mla7 + MlNo3, Mla10 + MlDu2, Mla13 + MlRu3*. By present many interesting findings on sources of resistance genes to barley powdery mildew have been published worldwide. Postulation of resistance genes to powdery mildew of barley is a major method of their identification in barley varieties. For instance, some researchers [1, 2, 24, 26, 28] postulated Ml-genes in winter and spring barley varieties by this method. Among them the following barley varieties are of particular interest for breeders in Kazakhstan: Algerian (Mla1) [1], Agra, Arve, Thule (sources of gene Mla9) [2, 28], Shamu, Baronesse (sources of gene Mla3) [2], Atlas (source of gene Mlat) [1], Kristaps, Rasa (sources of genes Mla7, MlNo3, Mlg) [28]. They are carriers of effective resistance genes to Kazakhstanean *Bgh* isolates. Besides experience from Europe suggests the best ways of achieving durable resistance is to use either *mlo* [29] or combinations of minor genes. The recessive resistance gene *mlo* has remained effective for more than 50 years and is the mainstay of mildew control in European winter barley plantings [5].

Minimal intrapopulation diversity judging on all statistical indices (*H*_*W*_, *H*_*G*_, *D*) was displayed by the population from Zhambyl region, and maximal one by the population from South Kazakhstan population (Table 5). The results of the study show significant difference even within the limits of one host variety. Spring barley variety Baisheshek is a source of infection in some areas of South Kazakhstan and Zhambyl regions (Table 2). Analysis of pathotype structure of the fungus isolated from this cultivar showed higher virulence diversity of South Kazakhstan isolates versus Zhambyl isolates. Since there is no information on resistance genes in commercial barley varieties it is impossible to assess reliably which genes could influence the results of the virulence analysis. Powdery mildew in Kazakhstan is mainly an infection that is introduced from other countries, so one of the factors causing this difference could be different ways of the colony migration. Primary infection could be brought into South Kazakhstan region by airflows from adjacent Central Asian countries (Uzbekistan, Tajikistan), and into Zhambyl region by airflows from Kyrgyzstan and China. Virulence of the pathogens from Zhambyl population actually does not differ from that of the isolates from Chinese population [22], and the structure of *Bgh* populations in Uzbekistan and Tajikistan is still not known. The greatest virulence polymorphism of isolates was observed on lines with genes *Mlra, Mlk, MlLa and Mlh* (from 10.0 to 78.6%).

The data of many studies show that the virulence of the barley powdery mildew pathogen differs greatly in different eco-geographical regions. For instance, Czech population demonstrates high phenotypical diversity and the lowest mean complexity of virulence versus Israel population [18]. Virulence of Chinese *Bgh* populations differs substantially from the same of European populations; the only similarity consists in virulence to genes *Mla8* and *Ml(Ch)* [22]. It has been recently shown that the structure of *Bgh* populations in South Africa is very variable, contains unique virulence frequencies and associations and differs from populations in other parts of the globe [21]. Comparison of our own results with published data displays low similarity of populations in Kazakhstan to European, African, Australian and South-West Asian populations. Particularly, the virulence rate of *Bgh* populations in Kazakhstan is much lower than in Europe [19, 20], South Africa [21] and South-West Asia [18]. The difference between Kazakhstanean and Chinese populations consists mainly in the fact that the pathogens isolated in South Kazakhstan are virulent to some effective resistance genes (*Mla3, Mlat, Mlg, Mla22*) in China [22]. The Australian population also differs from population in Kazakhstan, because the population of *Bgh* in our country contains the isolates virulent to gene *Mlra* that is effective in Australia [5].

## Conclusions

Differentiation of Kazakhstani barley powdery mildew population resulted in isolation and propagation of 23 pathotypes with different virulence to isogenic Pallas lines. Indices of diversity showed that virulence was very diverse within the pathogen population and the Gleason, Simpson and Shannon indices were high in South Kazakhstan region (*H*_*G*_ = 4.31, *D* = 0.89, *Sh* = 2.44) but low in populations from Zhambyl (*H*_*G*_ = 2.16, *D* = 0.78, *Sh* = 2.03). The predominant pathotypes 24 and 64 comprised 19.13–21.37% of the isolates from South Kazakhstan region and 10.78–14.19% of the isolates from Zhambyl region. High virulence (virulence complexity 5–6) is characteristic for unique pathotypes 72, 60 and 57 isolated from winter barley varieties. No isolate was virulent on differential lines possessing the resistance genes *Mla9, Mla1 + MlaAl2, Mla6 + Mla14, Mla13 + MlRu3, Mla7 + MlNo3, Mla10 + MlDu2, Mla13 + MlRu3* and *Mlo-5,* therefore these genes are effective in Kazakhstan. Besides high effectiveness to the most part of the agent pathotypes was demonstrated by *Mla3, Mlg + MlCP* and *Mla12 + MlEm2*. The use of sources of the above-mentioned genes ensures the successful breeding of barley for powdery mildew immunity. Since different selections of susceptible barley varieties are grown in Kazakhstan, micro-evolutionary changes of *Bgh* may result in emergence and propagation of new pathotypes within the pathogen population. The present study provides a foundation for future studies on the pathogenic variability within of *Bgh* populations in Kazakhstan and addresses the knowledge gap on the virulence structure of *Bgh* in Central Asia.

## Methods

### Location of pathogen populations

The areas under barley were surveyed in 11 districts of four regions of Kazakhstan and adjacent Omsk region (Russia) in 2015–2016 (Fig. 1). The pathogen was present only in seven districts of two regions (South-Kazakhstan and Zhambyl) of Kazakhstan (Figs. 1 and 2). Diseased samples were thus collected only from two regions within the surveyed districts in the period of high disease pressure from June to July. The distances between the survey regions ranged from 276 to 1155 km (Fig. 1), and between the sampling sites from 60 to 188.4 km (Fig. 2).

### Sampling populations and multiplication of inoculum

A total of 107 isolates were collected during the survey period with most of the isolates (57%) being collected in 2016 (Table 2). Among the two regions, the highest number of isolates was collected in South Kazakhstan (61.68%), followed by Zhambyl region (33.32%). The highest number of isolates was collected in the districts of Saryagash and Tulkibas in South Kazakhstan and Merke in Zhambyl region. Mainly the isolates have been collected from spring barley cultivar Baisheshek (49.53%) and winter cultivar Yuzhno-Kazakhstanskiy 43 (19.63%) that are admitted for cultivation in the Republic of Kazakhstan [30]. Tissue segments approximately 7 cm in length were excised from infected plants and placed in 100 mm plastic Petri dishes with 0.7% water agar and 35 ppm benzimidazole, then incubated for 1 day at 22 ± 2°C under artificial light (cool-white fluorescent lamps providing 12 h light at 15 ± 5 μmol m^−2^ s^−1^) [5, 21, 22]. Conidia from each colony were shaken onto leaf segments 25 mm long, which were excised from the central part of healthy, fully expanded primary leaves of the line B-3213 with no resistance gene to powdery mildew [31]. Inoculated leaf segments were placed in Petri dishes with water agar, prepared as above, and incubated under similar conditions for 10–11 days.

### Differential sets and inoculation of leaf segments

The set of differentials used in 2015–2016 (Table 1) was comprised of barley cv. Pallas and 16 near-isogenic ‘Pallas’ lines containing different genes for resistance to powdery mildew [10]. The differential set was kindly provided by Professor Mogens Støvring Hovmøller and Dr. Chris Khadgi Sørensen, Department of Agroecology, Aarhus University, Denmark.

About 50 seeds of each differential were sown in a pot filled with a gardening peat substrate and placed in a greenhouse under natural daylight for 12–14 days. Leaf segments 20 mm long were taken from the central part of healthy fully expanded primary leaves of each differential. Three leaf segments of each differential were placed with the adaxial side facing up in a 150 mm glass Petri dish on the above-mentioned water agar. Leaf segments of differentials were inoculated in a metal inoculation tower 415 mm high and 150 mm in diameter. For each isolate, a glass Petri dish with leaf segments from the differential set was placed at the bottom of the tower. Inoculum of each isolate collected from a leaf segment was shaken onto a square piece (40 × 40 mm) of black paper to estimate the number of conidia deposited, and blown through a hole of 15 mm diameter in the upper part of the inoculation tower. The dishes with inoculated leaf segments were incubated in a chamber at 18 ± 2 °C under artificial light (cool-white fluorescent lamps providing 12 h light at 30 ± 5 μmol m^−2^ s^−1^) [21, 22].

### Virulence determination

Reaction type (RT) on each differential and *Bgh* isolate combination was scored 8 days after inoculation on a 0–4 scale [32]. This scoring scale was supplemented with RT 0(4) (i.e., RT 0 with a few RT 4 colonies) [33], which is characteristic for barley lines carrying the *mlo* resistance gene. Isolates that produced RT 4 or 3–4 were considered virulent on the corresponding resistance gene(s). Virulence frequencies found during 2 years using seven individual populations are presented in Table 1. All tests for virulence of the Kazakhstanean populations were carried out at the Research Institute for Biological Safety Problems (Republic of Kazakhstan).

### Pathotype designation

The isolates were assigned numerical designations based on their virulence to matching resistance genes in six differentials (Nos. 1–6 in Table 1). The differential set was divided into two triplets and each of the two digits indicates virulence or avirulence on the three differentials of the respective triplet. If a virulence to the corresponding resistance gene is detected, the first differential line has the value 1 (2^0^), the second line has the value 2 (2^1^), and the third line has the value 4 (2^2^). Therefore, each digit can have a value from 0 (no virulence on any of the three differential lines) up to 7 (=1 + 2 + 4, virulent on each of the three differential lines). The resulting number based on six differentials defines the virulence of the isolates and consequently their classification as pathotypes [34, 35].

### Data analysis

Map depicting spatial locations of isolates were developed with the help of ArcGis 10.3 (ESI 2016) using coordinates of sites where infected samples were collected during the survey (Fig. 1). Parameters for comparison of all populations were calculated on the basis of virulence patterns of isolates on the set of six differentials in the given order. Descriptive parameters of populations (virulence frequency, virulence complexity, number of pathotypes, abundance and richness) were calculated for the isolates with the HaGiS program [36]. Abundance generally signifies the frequency of the predominant pathotype (%); and richness defines the number of pathotypes as a proportion of the number of isolates tested. In addition, *H*_*W*_ [37], *H*_*G*_ [38], *D* [39], and evenness (*E*) indices [40] were calculated to determine the diversity of virulence in the pathogen populations. These indices were calculated based on the number of isolates and *Bgh* pathotypes in each surveyed region and across all the seven districts surveyed during the study. These indices were calculated as follows:

1 $$ {H}_w=-\sum \left[{p}_i\ln \left({p}_i\right)\right] $$

where *p*_*i*_ is the frequency of the *i*th pathotype in population.

2 $$ {H}_G=\left(r-1\right)\mathit{\ln}(N) $$

where *r* is the number of distinct pathotypes and *N* is the total number of isolates in the population.

3 $$ D=1-\sum {\left({n}_i/N\right)}^2 $$

where *n*_*i*_ is the number of isolates representing the pathotype *i* and *N* is as defined above. As pathotype diversity increases, D approaches 1, while as pathotype diversity decreases, D approaches 0.

4 $$ E=H/{H}_{max} $$

where *H*_max_ is the maximal value of the diversity index with *E* ranging from 0 to 1, with *E* = 1 representing an equal abundance of all pathotypes.

## Abbreviations

*Bgh*: *Blumeria graminis* f.sp. *hordei*
*D*: Simpson index
*E*: Evenness
*H*_*G*_: Gleason index
*H*_*W*_: Shannon index
RT: Reaction type
